# Californium-252 neutron brachytherapy combined with external pelvic radiotherapy plus concurrent chemotherapy for cervical cancer: a retrospective clinical study

**DOI:** 10.1186/s40880-017-0191-x

**Published:** 2017-02-28

**Authors:** Shen Qian, Ling Ye, Yun-Hong Tian, Li-Gen Wang, Zuo-Ping Huang, Feng Li, Bing Hou, Ni Song, Juan Chen, Ying Liu, Xiao Liu, Tao Zhou

**Affiliations:** 1 Department of Oncology, Armed Police Hospital of Guangdong Affiliated with Guangzhou Medical University, No. 268 of Yanling Road, Tianhe District, Guangzhou, 510507 Guangdong P. R. China; 2 Department of Oncology, The First Affiliated Hospital of Jinan University, Guangzhou, 510630 Guangdong P. R. China; 3 Cancer Center of Guangzhou Medical University, Guangzhou, Guangdong 510095 P. R. China

**Keywords:** Cervical cancer, Californium-252, Neutron brachytherapy, External-beam radiotherapy

## Abstract

**Background:**

Cervical cancer is the sixth most common cancer in Chinese women. A standard treatment modality for cervical cancer is the combination of surgery, chemotherapy, external-beam radiotherapy and intracavitary brachytherapy. The aim of this study was to retrospectively assess the long-term treatment outcomes of patients with cervical cancer who were treated with californium-252 neutron brachytherapy combined with external-beam radiotherapy plus concurrent chemotherapy.

**Methods:**

We retrospectively analyzed the medical records of 150 patients with primary stages IB-IVB cervical cancer who received neutron brachytherapy combined with external-beam radiotherapy concurrently with cisplatin chemotherapy. All patients were followed up. Using an actuarial analysis, patient outcomes and treatment-related adverse effects were evaluated and compared.

**Results:**

The median overall survival (OS) was 33.2 months. The 3-year progression-free survival rates for patients with stages I–II, III, and IV diseases were 81.0% (68/84), 65.0% (39/60), and 0% (0/6), respectively; the 3-year OS rates were 90.5% (76/84), 85.0% (51/60), and 16.7% (1/6), respectively. Vaginal bleeding was controlled within the median time of 4.0 days. One month after treatment, 97.3% of patients achieved short-term local control. The local recurrence rates for patients with stages I–II, III, and IV disease were 4.8% (4/84), 11.7% (7/60), and 33.3% (2/6), respectively, and the occurrence rates of distant metastasis were 16.7% (14/84), 25.0% (15/60), and 100.0% (6/6), respectively. Cancer stage, tumor size, and lymph node metastasis were identified as prognostic risk factors, but only lymph node metastasis was found to be an independent prognostic factor. The most common adverse effects during treatment were grades 1 and 2 irradiation-related proctitis and radiocystitis.

**Conclusion:**

For patients with cervical cancer, neutron brachytherapy combined with external-beam radiotherapy plus concurrent chemotherapy produces a rapid response and greatly improves local control and long-term survival rates with tolerable adverse effects.

## Background

Cervical cancer is the sixth most common cancer in Chinese women [1]. In 2011, 87,982 cases of cervical cancer were reported, and the incidence was 13.40 per 10,000 women in China [1]. The treatment paradigms in the primary management of cervical cancer are well established, with early lesions being treated surgically or with brachytherapy alone [2] and locally advanced lesions being managed with concurrent chemoradiotherapy [3, 4]. The 5-year survival rate of patients with stage II or III disease who receive concurrent chemoradiotherapy ranged from 53% to 74%; however, the 5-year survival rate of patients with stage IV disease was only 20% to 30% [5]. Different treatment algorithms affect surviving women to varying degrees, and radiotherapy is associated with longer-term adverse effects than surgery or chemotherapy [6, 7]. Several studies have shown that adding brachytherapy to the treatment protocol results in prolonged survival for patients with cervical cancer [8–11]. Studies have also shown that nearly each radiotherapy protocol option for so-called radio-resistant tumors is more efficacious than photon brachytherapy; therefore, to improve the curability of cervical cancer, new types of radiation should be studied [12].

Californium-252 (^252^Cf) was first identified in the debris from a thermonuclear test explosion in 1950 [12]. The discovery raised the possibility of using fast neutrons in brachytherapy, with the neutron interaction being concentrated directly in the tumor tissues that need irradiation. It has been suggested that the resistance of tumor cells to gamma-ray radiation can be overcome by administering radiotherapy with neutron emitters [13, 14]. Several pilot studies have found that ^252^Cf brachytherapy for cervical cancer offers advantages over traditional gamma-ray radiation [12–16]. In the mainland of China, many patients with cervical cancer have been treated with ^252^Cf neutron brachytherapy (NBT) [17]. However, the widespread use of ^252^Cf NBT in the mainland of China has been limited by insufficient clinical data regarding its efficacy and safety.

In this retrospective clinical study, we analyzed the long-term survival, adverse effects, metastasis, and recurrence in 150 patients with cervical cancer who were treated in 2010 with ^252^Cf NBT combined with external-beam radiotherapy (EBRT) plus concurrent chemotherapy at the Armed Police Hospital of Guangdong Affiliated with Guangzhou Medical University.

## Methods

### Patient population

Between January 2010 and December 2010, more than 400 patients with primary cervical cancer were treated with concurrent chemotherapy combined with ^252^Cf NBT and EBRT at the Armed Police Hospital of Guangdong Affiliated with Guangzhou Medical University, Guangzhou, China. The patients with newly diagnosed cervical cancer and without previous treatment were selected. Patients with any of the following conditions were excluded: (1) bone marrow suppression (peripheral blood leukocyte count <3 × 10^9^/L and platelet count <7 × 10^9^/L); (2) uncontrolled acute or sub-acute pelvic inflammatory disease; (3) extensive tumor, qualitative cachexia, or uraemia; (4) acute hepatitis or uncontrolled severe cardiovascular disease; and (5) pregnancy, a history of malignancies, or previous treatment with chemotherapy, surgery, or radiotherapy.

Cervical cancer was staged according to 1997 International Federation of Gynecology and Obstetrics recommendations. Prior to treatment, all patients underwent physical examination, gynecologic examination, chest computed tomography (CT), electrocardiography, and abdominal and urinary system B ultrasound. Also prior to treatment, tumor size and lymph node status were determined with magnetic resonance imaging (MRI) or CT. Lymph node metastasis was defined as the status that lymph node exhibited loss of oval shape and were greater than 1.0 cm in diameter. The Academic Committee of the Armed Police Hospital of Guangdong Affiliated with Guangzhou Medical University approved this study.

### ^252^Cf NBT

In the Department of Oncology at the Armed Police Hospital of Guangdong Affiliated with Guangzhou Medical University, ^252^Cf intracavitary after-loading NBT was performed using an LZH-1000 NBT instrument (Model ZH-1000; Linden Science and Technology, Shenzhen, Guangdong, China). The ^252^Cf neutron source of this unit was 712.787–503.881 µg with a half-life of 2.65 years, involving a decay process that launches neutron and gamma rays and has a neutron emission rate of 2.3 million neutrons per second, an average energy of 2.14 MeV (gamma rays), an average energy of 0.8 MeV (neutron rays), and a neutron relative biological effect (RBE) of 2–3 [18]. The radioactive source used for the patients included in the present study was changed in 2010.

**Fig. 1:**
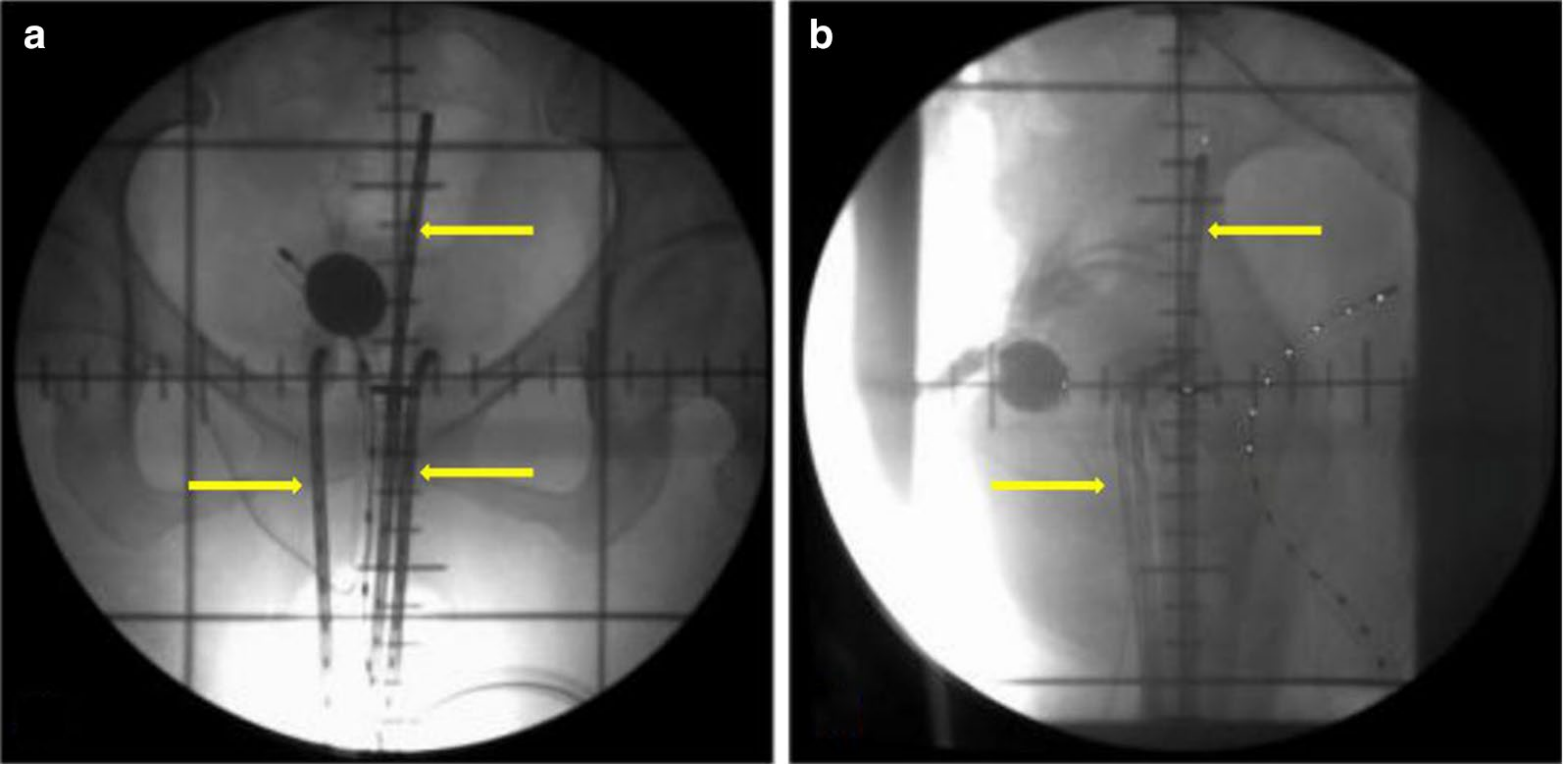
Schematic representation of californium-252 (^252^Cf) neutron brachytherapy (NBT). Contrast media was injected into a Foley catheter. Then, three passive cavity applicators were inserted into the uterine cavity and bilateral fornix; the applicator that delivers ^252^Cf was inserted and fixed to the vagina and uterine cavity. Finally, orthogonal radiographs of the pelvic cavity were acquired from the antero-posterior (**a**) and lateral (**b**) directions. The *yellow arrows* indicate the cavity applicators

Prior to the administration of ^252^Cf NBT, the patients received a rectal barium enema to visualize the rectum, and 7 mL of contrast media was injected into a Foley catheter through a urine catheter inserted into the bladder. A vaginal and rectal bimanual triple diagnosis was obtained by observation of the dilated cervix and vagina. Then, three passive cavity applicators were inserted into the uterine cavity and bilateral fornix, subsequently, the vagina was tight packing with gauze. According to the tumor size, the applicator was inserted after routine sterilization and was positioned and fixed to the vagina and uterine cavity. After the applicator was fixed, orthogonal radiographs of the pelvic cavity were acquired to display the position of the applicator and the extent and size of the tumor (Fig. 1). Dosimetry was prescribed to “point A,” defined by the Manchester system as 2.0 cm superior (along the tandem) to the flange abutting the external cervical os and 2.0 cm lateral from the axis of the tandem.

Correspondingly, the doses were calculated at standardized point B, which is located at 5.0 cm from the uterine central axis and represents part of the obturator lymph nodes as well as the bladder and rectal points.

The dose of ^252^Cf was calculated in Gy-equivalents (Gy-eq) according to the following formula:

$${\text{D }}\left( {{\text{Gy}} - {\text{eq}}} \right) = {\text{Dn}} \times {\text{RBEn}} + {\text{D}} \gamma \times {\text{RBE}}\gamma$$

where Dn is the dose of the neutron component of ^252^Cf, Dγ is the dose of the gamma-ray component of ^252^Cf, RBEn is the relative biological effectiveness of the neutrons, and RBEɣ is the relative biological effectiveness of the gamma rays.

The RBE of the neutron component of ^252^Cf for the tumor tissue was estimated to be 6 [18–20]. The doses at point A were calculated assuming a high-dose-rate delivery of 6 Gy-eq for each patient per fraction, administered once per week, to deliver a total dose of 30 Gy. Optimized treatment plans were based on tumor size; the exposure of the bladder and rectum was no more than 60% of the dose at point A.

### External pelvic irradiation

The use of CT-based treatment planning and conformal blocking was considered the standard methods for EBRT. CT scans of the pelvic cavity were taken to determine the extent and size of the tumor. The external-beam radiation administered to the patients was produced by standard X-ray beams generated from a medical linear accelerator with an energy level of 8 MeV (Elekta, Stockholm, Sweden).

The applied dose was delivered at two opposite fields as divided doses to full and split fields of the pelvis, extending to the para-aortic lymph nodes. The anterior edge of the lateral fields included the external iliac lymph nodes, and the posterior edge included the presacral lymph nodes. In general, the upper boundary was the fifth lumbar vertebra; however, for patients with documented common iliac and/or para-aortic nodal involvement, the upper boundary reached the level of the renal vessels. The lower boundary was located on the lower edge of the obturator or ischial tuberosity, and the left and right boundaries were 1.5–2.0 cm beyond the outside of the pelvis. The typical size ranged from 15.0 to 17.0 cm. For patients with negative lymph nodes (i.e., the lymph nodes without metastasis as determined by CT and/or MRI scans), the irradiation volumes included the gross primary cervical tumor; the uterus; the paracervical, parametrial, and uterosacral regions; and the entire external iliac, internal iliac, and obturator nodal basins. For patients with bulk tumors (diameter >4.0 cm) or suspected nodes confined to the low pelvis, the irradiation volume was extended to cover the common iliacs. For patients with documented common iliac and/or para-aortic nodal involvement, the irradiation field was extended to the level of the renal vessels. The prescribed radiation dose for the clinical target was a daily fraction of 2.0 Gy, administered 5 days per week; a variation of up to 5% was considered acceptable. The total dose administered to the whole pelvis was 45–50 Gy. For patients with bulk tumors, the parametrial disease area was further irradiated with an additional boost of 8–10 Gy in 4–5 fractions, and the dose to the gross pelvic nodes reached 60–65 Gy.

Above all, the total target doses administered to all patients during the treatment were 85 Gy-eq at point A and approximately 60 Gy-eq at point B. In other words, EBRT was initiated (1.8–2.0 Gy/day) for the total dose at point A. Then, NBT at a total dose of 30 Gy-eq was delivered. The period during the treatment with 8-MV X-ray EBRT and ^252^Cf NBT was 6 weeks.

### Amifostine application

To prevent irradiation-related enteritis, all patients received 2 mg (20 mL) amifostine, dissolving in saline to a concentration of 50 mg/mL, in the rectum for 30–45 min once per week [21]. To avoid infection and vaginal perineum contracture, the patients underwent perineal douche every day during the radiotherapy for the pelvis but not on the day of ^252^Cf NBT.

### Concurrent chemotherapy

During pelvic EBRT, all 150 patients received 40 mg/m^2^ cisplatin on days 1, 8, 15, 22, 29 and 36. Prior to chemotherapy, anti-emetic drugs, preventive measures, and hydration treatments were administered to alleviate chemotherapy-related symptoms. Blood tests, blood biochemistry assessments, and gynecologic examinations were performed weekly to determine whether the lesions had regressed and to evaluate the clinical situation.

### Evaluation and follow-up

After completion of therapy, all patients were followed up 1 month after treatment, then every 3 months up to 1 year, and thereafter every 6 months up to 3 years through outpatient department visits. The patients lost to follow-up were classified as censored. The follow-up included routine physical examination, blood cell counts, pelvic examination, MRI of the abdomen and pelvis, chest X-ray radiography, abdominal B ultrasound, cervical/vaginal cytology, and row biopsy for residual or recurrent local cervical tumors.

According to World Health Organization standards related to therapeutic effects in solid tumors, short-term local curative effects observed within 1 month after treatment were categorized as complete response (CR), partial response (PR), stable disease (SD), and progressive disease (PD). For the patients with CR and PR, the treatment was considered effective. During the first month after treatment, the tumor masses in the abdomen and pelvis were evaluated through MRI. Progression-free survival (PFS) was calculated from the date when radiotherapy was administered to the date of local recurrence, distant metastasis, death, or the last follow-up. Overall survival (OS) was calculated from the date when the treatment of the patients was administered to the date of death or the last follow-up. The irradiation-related adverse effects were graded according to the 1995 Radiation Therapy Oncology Group/European Organization for Research and Treatment of Cancer (RTOG/EORTC) guidelines. RTOG acute radiation morbidity criteria were used for the evaluations conducted during irradiation or within the first 90 days after treatment. RTOG late radiation morbidity criteria were used to evaluate adverse effects observed after completion of irradiation. Toxicity classified as grade 3 or higher was considered severe. The irradiation-related adverse effects included bone marrow suppression, gastrointestinal reactions, irradiation-related enteritis, intestinal obstruction, intestinal fistula, irradiation-related cystitis, and urinary fistula [7, 9, 13, 15].

### Statistical analysis

All data were analyzed using SPSS 17.0 software (SPSS, Inc., Chicago, IL, USA). Kaplan–Meier plots were used for survival analysis and Cox regression was used for the multivariate analysis. Unless otherwise stated, all reported *P* values were two-tailed. *P* values less than 0.05 were considered statistically significant.

## Results

### Patient characteristics

We included 150 patients with cervical cancer in this retrospective study. The patients ranged in age from 27 to 79 years (median, 50 years). Other patient characteristics are listed in Table 1.

**Table 1:** Characteristics of 150 patients with cervical cancer

Characteristic	No. of patients (%)
Stage (FIGO)	
I	2 (1.3)
IA	0 (0.0)
IB	2 (1.3)
II	82 (54.7)
IIA	13 (8.7)
IIB	69 (46.0)
III	60 (40.0)
IIIA	15 (10.0)
IIIB	45 (30.0)
IV	6 (4.0)
IVA	4 (2.7)
IVB	2 (1.3)
Histopathologic type	
Squamous cell carcinoma	146 (97.3)
Adenocarcinoma	3 (2.0)
Clear cell carcinoma	1 (0.7)
Pre-treatment tumor size (cm)	
≤4.0	85 (56.7)
>4.0	65 (43.3)
Lymph node metastasis	
Yes	49 (32.7)
Not	73 (48.7)
Unknown	28 (18.6)

*FIGO* International Federation of Gynecology and Obstetrics

### Control of vaginal bleeding

Of the 150 included patients, 116 (77.3%) exhibited symptoms of vaginal bleeding, including 2 (1.3%) with stage I cervical cancer, 35 (23.3%) with stage II disease, 73 (48.7%) with III disease, and 6 (4.0%) with stage IV disease. This irregular vaginal bleeding was controlled within 3–6 days (median, 4 days). Because very few patients had stage I disease, we combined the patients with stage I and stage II disease into one group for data analysis.

### Short-term local curative effects

All 150 patients were followed up 1 month after treatment. After all 150 patients underwent ^252^Cf NBT combined with EBRT plus concurrent chemotherapy, 138 (92.0%) reached CR, 8 (5.3%) reached PR, 4 (2.7%) had stable disease, and none had PD. Overall, ^252^Cf NBT resulted in a short-term local effective rate (CR + PR) of 97.3%. Analysis of patients with a tumor ≤4.0 cm in diameter showed a reduction in tumor mass to 50% of the original volume after one cycle of ^252^Cf NBT. It took 5–16 days (median, 9.5 days) for these patients to achieve PR. For patients with a tumor >4.0 cm in diameter, the tumor began to shrink after two cycles of ^252^Cf NBT. It took 5–23 days (median, 18 days) for these patients to achieve a CR or PR state.

### Survival

**Fig. 2:**
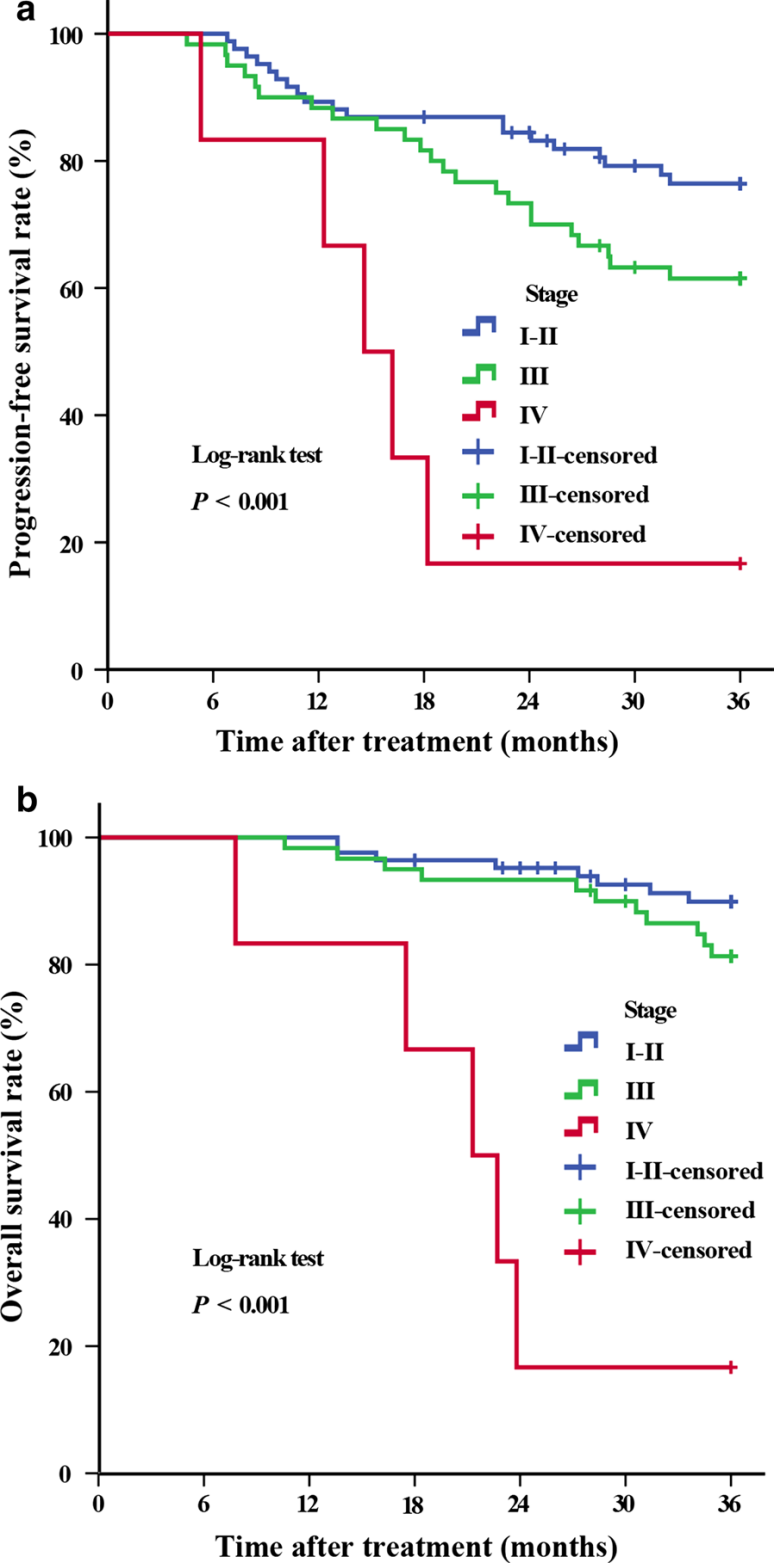
Kaplan-Meier curves of 3-year progression-free survival and 3-year overall survival for 150 patients with cervical cancer who were treated with ^252^Cf NBT combined with external-beam radiotherapy (EBRT) plus concurrent chemotherapy. **a** Progression-free survival; **b** overall survival

During the 3-year follow-up period, 12 patients were lost to follow-up and 24 patients died. Of the 24 patients who died before the last follow-up, 2 died within 1 year after the first treatment, 11 died in the second year, and 11 died in the third year. The 3-year median overall survival time was 33.2 months (range, 7.8–36.0 months). The PFS rates for patients with stages I–II, III, and IV cervical cancer were 81.0% (68/84), 65.0% (39/60), and 0% (0/6), respectively; and the 3-year OS rates for these patients were 90.5% (76/84), 85.0% (51/60), and 16.7% (1/6), respectively (Fig. 2).

### Factors related to the prognosis of patients with cervical cancer

Univariate analysis showed that stage, tumor size, and lymph node metastasis were factors that significantly affected prognosis (Table 2). However, Cox analysis showed that only lymph node metastasis was an independent prognostic factor (Table 3).

**Table 2:** Univariate analysis of factors related to 3-year overall survival rate of cervical cancer patients

Characteristic	*P* value	HR	95% CI
Stage (FIGO)	<0.001		
III vs. I–II	0.181	1.95	0.74–5.13
IV vs. I–II	<0.001	20.60	6.11–69.39
Pre-treatment tumor size (≤4.0 cm vs. >4.0 cm)	<0.001	0.17	0.06–0.4
Lymph node metastasis (yes vs. no)	<0.001	0.02	<0.01–0.17
Age (per 1-year increase)	0.850	1.01	0.96–1.05

Patients with unknown lymph node status were excluded from the univariate analysis with the characteristic of lymph node metastasis

*HR* hazard ratio, *CI* confidence interval, *FIGO* International Federation of Gynecology and Obstetrics

**Table 3:** Multivariate analysis results for overall survival rate of cervical cancer patients using the Cox multiple regression method

Characteristic	*P* value	HR	95% CI
Stage (FIGO)			
III vs. I–II	0.398	0.63	0.21–1.85
IV vs. I–II	0.271	2.14	0.55–8.28
Pre-treatment tumor size (≤4.0 cm vs. >4.0 cm)	0.908	3.11	0.82–7.90
Lymph node metastasis (yes vs. no)	0.001	36.32	4.65–283.92

Patients with unknown lymph node status were excluded from the multivariate analysis with the characteristic of lymph node metastasis

*HR* hazard ratio, *CI* confidence interval, *FIGO* International Federation of Gynecology and Obstetrics

### Local recurrence and distant metastasis

The cancer recurrence rates in the pelvis for patients with stages I–II, III, and IV diseases were 4.8% (4/84), 11.7% (7/60), and 33.3% (2/6), respectively. The occurrence rates of distant metastasis for patients with stages I–II, III, and IV diseases were 16.7% (14/84), 25.0% (15/60), and 100% (6/6), respectively. All 24 patients who died had recurrence or metastasis within 3 years; of these patients, 3 experienced single local recurrence, and 21 had distant metastasis. Among the 24 patients who died, 18 had distant metastasis in the bone, 18 in the left supraclavicular lymph nodes, 17 in the lung, 17 in the para-aortic lymph nodes, 16 in the liver, and 12 in the inguinal nodes.

### Acute and late adverse effects

We retrospectively analyzed the occurrence rate of acute and late irradiation-related adverse effects in the 150 patients with cervical cancer. Immediately after treatment, 11 (7.3%) experienced irradiation-related proctitis, with symptoms of tenesmus, down-bearing distention of the anus, and diarrhea; 6 patients (4.0%) experienced acute radiocystitis, with symptoms of increased urinary frequency and urgency and pain while urinating; and 1 (0.7%) experienced acute hematuria. The reactions and late adverse effects observed after NBT are presented in Table 4. Late adverse effects primarily occurred in the intestinal tract and urinary system. According to RTOG/EORTC scoring criteria, the most common adverse effects were irradiation-related proctitis and radiocystitis.

**Table 4:** Irradiation-related adverse effects in 150 patients with cervical cancer who were treated with californium-252 neutron brachytherapy combined with external-beam radiotherapy

Adverse effect	Total	Grade I	Grade II	Grade III	Grade IV
Irradiation-related proctitis	150 (100)	134 (89.3)	13 (8.7)	2 (1.3)	1 (0.7)
Radiocystitis	148 (98.7)	130 (86.7)	11 (7.3)	7 (4.7)	0 (0.0)
Rectovaginal fistula	3 (2.0)	NA	NA	NA	NA
Down-bearing distention of the anus	75 (50.0)	NA	NA	NA	NA

All values are presented as number of patients followed by percentage in parentheses

*NA* not applicable

## Discussion

Our retrospective study assessed the long-term treatment outcomes of cervical cancer patients treated with ^252^Cf NBT combined with EBRT plus concurrent chemotherapy. We found that, for the patients with stages I–II, III, and IV diseases, the PFS rate was 81.0%, 65.0% and 0%, respectively; the 3-year OS rate was 90.5%, 85.0% and 16.7%, respectively; the local recurrence rate was 4.8%, 11.7% and 33.3%, respectively; and the occurrence rate of distant metastasis was 16.7%, 25.0% and 100%, respectively. The most common adverse effects during treatment were irradiation-related proctitis and radiocystitis.

For over 100 years, brachytherapy has been an essential component in the treatment of cervical cancer [4], and gamma ray-emitting sources, such as cobalt-60 (^60^Co), iridium-192 (^192^Ir), and caesium-137 (^137^Cs), are widely used in brachytherapy [17]. These radionuclides create low-linear energy transfer (LET) radiation with poor efficacy against hypoxic tumor cells [22]. Tumor cells are resistant to conventionally fractionated irradiation because they present a high degree of DNA damage repair, but these cells are not resistant to high-LET irradiation (such as neutron radiation), which kills the cells with single-hit, non-repairable damage [23]. NBT is a form of high-LET radiotherapy that has been shown to be effective in killing radio-resistant cancer cells [5, 7]. In 1979, Maruyama et al. [20] published the first therapeutic results of the application of ^252^Cf NBT combined with external gamma-ray radiation for the intracavitary treatment of cervix cancer. Several studies have shown that ^252^Cf NBT, as a treatment of cervical cancer, is significantly more effective than conventional low-LET irradiation [14, 18, 21]. Janulionis et al. [24] conducted a long-term retrospective study comparing the outcomes of ^252^Cf and ^60^Co isotope intracavitary brachytherapy combined with brachytherapy in the treatment of patients with stage IIB cervical cancer and found similar OS rates in both treatment groups. However, the rate of tumor recurrence was significantly lower in the ^252^Cf group than in the ^60^Co intracavitary brachytherapy group. Tacev et al. [15] retrospectively studied the curative rate of patients with stages IIB and IIIB cervical cancer after ^252^Cf NBT supplemented with radium-226 (^226^Ra) or ^137^Cs intracavitary brachytherapy or with only gamma-ray radiation (^226^Ra or ^137^Cs); they observed significantly improved 5-year survival rates, regardless of tumor stage, for patients treated with ^252^Cf NBT compared with those who received conventional treatment. One trial of 206 patients with stage III cervical cancer who were treated with gamma-ray irradiation reported the 3-year OS rate ranging from 66.3% to 69.6%, which is lower than that found in our study (85.0%) [25]. A clinical study of stage IIB cervical cancer patients who were treated with low dose rate ^192^Ir intracavitary brachytherapy found that the response to radiotherapy was a strong predictor of local control, with 82% of patients continuing to have local control of the pelvis after the initial CR [26].

However, the local control rate (82%) reported by Budrukkar et al. [26] was lower than that in our study (97.3%). In another study, 32 patients with mainly advanced-stage squamous cell carcinoma received gamma-ray brachytherapy, and the 5-year OS, PFS, and local control rates were 75%, 68.5% and 92.8%, respectively [27]. The above-mentioned findings and those obtained in our study showed the advantage of the neutron source of ^252^Cf in brachytherapy for cervical cancer, mainly by overcoming tumor resistance to conventional photon irradiation; in particular, ^252^Cf NBT combined with EBRT was superior in terms of local control rate. The superiority of the neutron source of ^252^Cf in brachytherapy for cervical cancer may be attributed to the fact that high-LET irradiation causes irreversible changes in cellular systems [18, 28, 29] and that the ^252^Cf neutron ray demonstrates a high-energy deposition rate and accurate positioning, exhibits only a small dependence on oxygen, and results in only slight damage to normal tissue [30]. As a result, ^252^Cf neutron NBT is superior to traditional gamma ray-emitting sources used in intracavitary brachytherapy for cervical cancer.

In patients with cervical cancer, locoregional recurrence and distant metastasis are the major causes of clinical failure, followed by local-distant recurrence and para-aortic lymph node metastasis. Patients with recurrent lesions in the irradiation field may experience central or peripheral recurrence. However, once patients develop distant metastases, their prognoses are very poor. In our present study, 24 patients died within 3 years, and the most common sites of metastasis were the lung, bone, and liver.

In this study, univariate analysis showed that tumor size, lymph node metastasis, and tumor stage were associated with OS. However, Cox analysis showed that only lymph node metastasis was an independent prognostic factor. We did not find a significant association of pathologic type with patient prognosis because the vast majority (146 of 150) of patients had squamous cell carcinoma and only 4 patients had adenocarcinoma or clear cell carcinoma. Therefore, additional larger-scale clinical data analysis is necessary.

In this study, the adverse effects of ^252^Cf NBT were mainly irradiation-related proctitis and cystitis; specifically, the most common occurrence rates of grade 1 irradiation-related proctitis and cystitis were 89.3% and 86.7%, respectively. The short-term adverse effects associated with ^252^Cf NBT include vaginal stenosis, stenosis of the rectum, and rectovaginal fistula. Of the 150 patients included in this study, 3 (2.0%) developed rectovaginal fistulas. Our study showed that the adverse effects that patients with cervical cancer who underwent ^252^Cf NBT experienced were minimal and tolerable. Patients with grade 1 to 2 adverse effects required either no treatment or simple outpatient management. Patients with moderate to severe morbidity (RTOG grades 3–5), who experienced symptoms that were distressing enough to reduce their quality of life, required more intense management of adverse effects. These adverse effects might have been related to the long duration of urethral catheter placement during each treatment (because the neutron source was in its second half-life, each treatment lasted 3 h).

To reduce the occurrence rate of irradiation-related enteritis and cystitis, we administered amifostine as a rectal suspension once per week. Several studies have shown that amifostine protects various cells from irradiation damage by removing oxygen radicals and detoxifying the reactive metabolites of cytotoxic agents [21, 31, 32]. Furthermore, amifostine induces endothelial cell proliferation and the ensuing neovascularization, which contributes to wound healing [33, 34]. It is important to maintain the proper distance between the vagina and the bladder in the anterior direction and the proper distance between the vagina and the rectum in the posterior direction. In a study of patients undergoing high-dose-rate ^192^Ir intracavitary brachytherapy, Ferrigno et al. [35] reported that the 5-year occurrence rates of late adverse effects in the rectum, bladder, and small bowel were 16%, 11% and 14%, respectively. Compared with these data, the occurrence rates of late adverse effects in the present study were markedly lower (Table 4).

Since 1999, concurrent chemotherapy with irradiation has been the standard treatment of cervical cancer [36, 37]. For patients with stage IIB and more advanced cervical cancer, the results of many randomized clinical studies on the use of synchronous chemotherapy and radiotherapy showed that these treatments significantly improved local control and survival rates and that irradiation-related adverse effects were tolerated [37].

Our study did have several limitations. First, the database of the irradiation-related adverse effects was not very comprehensive and detailed because of the retrospective nature of the study. Second, this study did not include a control group that was treated with a different type of radiation. Third, our clinical data regarding cervical cancer treatment using ^252^Cf NBT were obtained from a single center. Multicenter trials should be conducted to further standardize treatment programs. Additional studies are needed to determine the optimal fractionation schedule of ^252^Cf NBT as well as optimal approaches to reduce the adverse effects of radiotherapy and increase tumor response to radiotherapy, thereby improving the prognosis of patients with cervical cancer.

## Conclusions

In summary, ^252^Cf NBT combined with EBRT plus concurrent chemotherapy as a treatment of cervical cancer offers a highly effective therapeutic modality with tolerable adverse effects and can improve the local control and long-term survival rates of patients. OS of cervical cancer patients treated with ^252^Cf NBT combined with EBRT plus concurrent chemotherapy was associated with tumor size, tumor stage, and lymph node metastasis.
